# Hospital Ethical Climate and Job Satisfaction among Nurses: A Scoping Review

**DOI:** 10.3390/ijerph19084554

**Published:** 2022-04-10

**Authors:** Patrycja Ozdoba, Magdalena Dziurka, Anna Pilewska-Kozak, Beata Dobrowolska

**Affiliations:** 1 Department of Holistic Care and Management in Nursing, Faculty of Health Sciences, Medical University of Lublin, 20-081 Lublin, Poland; md.dziurka@gmail.com (M.D.); beata.dobrowolska@umlub.pl (B.D.); 2 Department of Obstetrics and Gynecology Nursing, Faculty of Health Sciences, Medical University of Lublin, 20-081 Lublin, Poland; annapilewskakozak@umlub.pl

**Keywords:** ethical climate, job satisfaction, nurses, scoping review, work

## Abstract

The aim of this study was to map and summarize the published research findings on hospital ethical climate and its relationship with nursing staff job satisfaction as well as strategies proposed in the literature for the improvement of hospital ethical climate and job satisfaction through the actions of nursing staff in leadership positions. A scoping review has been performed in accordance with the guidelines of Preferred Reporting Items for Systematic Reviews and Meta-Analysis extension scoping reviews statement (PRISMA-ScR). Three electronic bibliographic databases were searched: the SCOPUS, Medline, and CINHAL Complete using a combination of keywords with the range of years 1994–2021. A total of 15 papers out of 235 records identified were eligible for the analysis. The literature review confirmed a significant relationship between ethical climate and job satisfaction of nurses. Furthermore, the interdependence of ethical climate and job satisfaction of nursing staff affects many different aspects including patients, co-workers, an organization and research. Identifying factors that influence ethical climate and job satisfaction as well as the relationship between these variables may help to reduce the dropout concerning a change of profession among nursing staff.

## 1. Introduction

Ethical climate can be defined as a set of behaviors, emotions and impressions characteristic for a given organization and shaped by a number of factors, such as professional values, norms, views, and cultivated tradition. The concept of the Ethical Climate Theory (ECT) dates back to the 1980s. The ECT authors, B. Victor and J.B. Cullen, classified the following five types of climate: caring, independent, rules, rights referred to as professional, and instrumental [1]. The term ethical climate was redefined by referring to nursing staff perceptions of how ethical issues are addressed in their specific work environment [2]. The level of ethical climate is largely determined by the relationships between nurses and colleagues, patients, physicians, managers, and the hospital [3]. Ethical climate is the foundation upon which professional nursing delivery is based [4].

Job satisfaction is a feeling of pleasure or lack of satisfaction with one’s job duties shaped by many factors including salary, working conditions, work experience, personal development, and many others closely related to the ethical climate of the institution where the employee is employed [5].

Over the past two decades, there has been a growing interest among researchers in the concept of ethical climate in the nursing environment and more specifically, its relationship with job satisfaction [6]. Research in this area is being conducted worldwide, namely in Iran [5,7,8], Ethiopia [9], South Korea [10], Turkey [11,12], Finland [13], Egypt [14], Taiwan [15,16], Israel [17], Bosnia and Herzegovina [18], and the USA [19,20].

Two questionnaires are mainly used to assess the level of ethical climate: the Ethical Climate Questionnaire (ECQ) compiled by Victor and Cullen in 1988 and the Hospital Ethical Climate Survey (HECS) created by Olson in 1995. Both of these tools are validated and show good psychometric properties: Cronbach’s alpha for ECQ is 0.86 to 0.92, and for HECS is 0.91 [3,21]. Job satisfaction is assessed by means of the Minnesota Job Satisfaction Questionnaire (MSQ [22], Index of Job Satisfaction (JS) [23], the Job Satisfaction Index [24], and the Manual for the Managerial Job Satisfaction Questionnaire [25], which have shown to have acceptable reliability and validity.

According to Victor and Cullen’s conception, the instrumental climate promotes the mission of the organization but is focused on maximizing the interest of individual employees [1]. This translates into a highly competitive work environment; therefore, this type of climate is the least desirable in a health care unit [7]. The caring type of climate is characterized by concern for others and consideration of their needs, which fosters teamwork supporting organizational effectiveness through decision making that should reflect maximum benefit to all stakeholders [16]. The climate of rules or principles encourages adherence to policies and procedures that outline the organization’s norms and expectations for the personnel employed. The creation of internal regulations is intended to improve decision making as this remains in the interest of the organization. In the climate of independence, employees can be expected to follow their personal and moral beliefs. Based on this, each individual decides for themselves what is right and wrong [16]. In contrast, the climate of rights and codes, also known as the climate of professionalism, refers to the fundamental criterion of ethics. For this climate, the first issue to consider is whether the decision made by a nurse/midwife is a violation of laws and codes. Nurses and midwives are required to strictly adhere to the law, professional standards, and code of ethical professional behavior as the fundamental issue of the profession [17].

A different approach to the concept of ethical climate is presented by Linda Olson, who defines it as individuals’ perceptions of organizational actions regarding ethical decision making and considerations that include aspects of power, trust, and interpersonal relationships within the organization [3]. On this basis, five types of relationships have been distinguished that significantly affect the ethical climate in the work environment and they regard relationships with colleagues, patients, managers, doctors, and the hospital.

Factors that shape the ethical climate of a health care unit include leadership styles and behaviors that foster ethical choices and actions beginning at the highest level of management. Thus, the key role is played by the leader who creates standards of conduct for an ethical climate in nursing practice [2]. Elements of the organization such as its history, mission, vision, and value system are reflected in various measures of ethical climate. Ethical climate in itself is not static because it undergoes dynamic changes depending on the aforementioned factors [2].

There are many studies on ethical hospital climate [1,2,3,4] and many empirical studies showing the relationship between ethical hospital climate and nurses job satisfaction [5,6,7,8,9,10,11,12,13,14,15,16,17,18,19,20]. Therefore, it is worth summarizing the current state of knowledge and identifying future research directions and practical actions in this field. Therefore, the aim of this scoping review is to map and summarize the current research findings on hospital ethical climate and its correlation with nursing staff job satisfaction, as well as to analyze proposed strategies for improving hospital ethical climate and job satisfaction through the actions of medical staff in leadership positions.

## 2. Materials and Methods

### 2.1. Design

**Figure 1:**
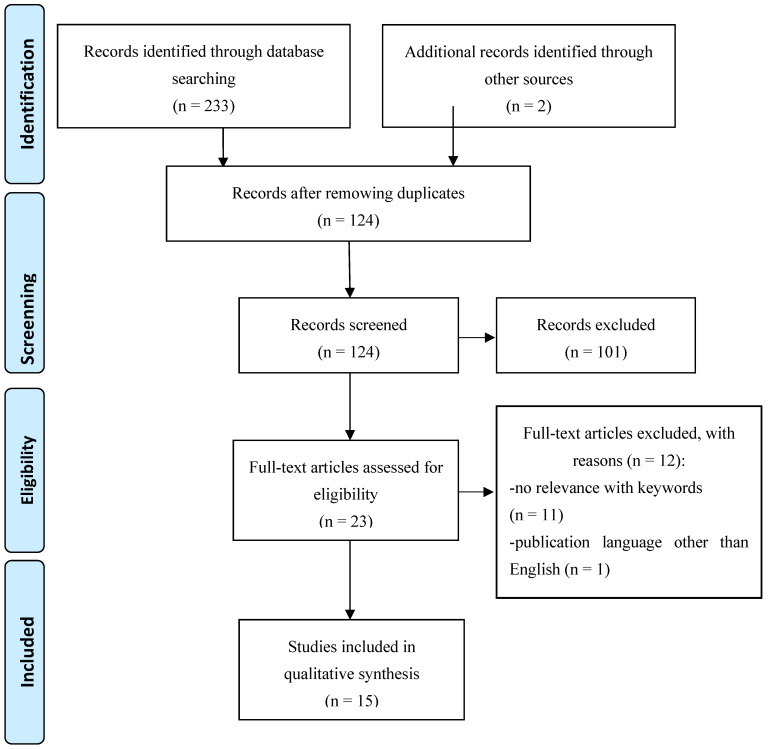
Flow diagram of search and selection process of scoping review.

A scoping review of the literature was conducted according to the principles and guidelines of the Preferred Reporting for Systematic Reviews and Meta-Analysis Extension for Scoping Reviews (PRISMA-ScR) that enabled the identification of available scientific evidence on the specific research issue and existing gaps or major assumptions and theories [26]. This is shown in Figure 1—flow diagram of search and selection in the scoping review process.

The analysis of the research material followed a methodological framework including a five-step approach by Arksey and O’Malley to the conduction of a scoping literature review [27]: (1) identification of the research question; (2) identification of relevant studies; (3) selection of studies; (4) data charting; and (5) results collation, summary, and report.

### 2.2. Research Questions

The following research questions were formulated:

1. What knowledge does the literature provide about the correlation between hospital ethical climate and nurses’ job satisfaction, and in what dimensions does this correlation occur?
2. What actions result from the studies analyzed to improve hospital ethical climate and nurses’ job satisfaction?

### 2.3. Studies Identification

A review of the literature available in electronic databases (Medline and CINAHL Complete (EBSCO Host) and SCOPUS) from 1994 to February 2021 was performed.

The initial date of the period was chosen because it is the beginning of ethical climate research. In addition, it is important to note how methods, instruments, results, and overall approaches to the research issue have changed over the years. The following combination of keywords was used ‘hospital ethical climate’, ‘ethical climate’, ‘job satisfaction’, ‘nurses’, ‘work’ combined with an AND operator.

A total of 15 papers out of 235 were qualified according to the inclusion criteria concerning articles published in English, empirical studies (primary data coming from qualitative, quantitative, and mix-methods studies), availability of the abstract and full version of the paper. Exclusion criteria included books and chapters in books, dissertations, and letters to the editor, review papers, meta-analyses, and systematic reviews.

### 2.4. Studies Selection

A literature review including article titles and abstracts was conducted independently by four researchers, and the results were analyzed and discussed by them. At the next stage, the full content of the eligible papers from the first stage was assessed independently which resulted in the final list of publications included in the scoping review analysis.

### 2.5. Data Charting

The eligible literature was selected to follow the data such as author(s), year of publication, country, purpose(s) of the study, method(s), research tools, study group, and the main results relevant to the research questions posed.

### 2.6. Results Collation, Summary, and Report

On the basis of the two research questions formulated, an in-depth analysis was carried out according to their essential features. Its results were presented in a descriptive manner. An inductive thematic analysis of the data contained in the literature considered for the study was performed. The most important information with basic numerical analysis of the nature and distribution of the studies included in the scoping review, specifically regarding methods, the group studied, and the main conclusions of the results of the work are presented in Table 1.

## 3. Results

A total of 233 articles were identified by searching databases and 2 publications from other sources. A total of 111 duplicate papers and 101 papers did not meet the inclusion criteria so they were excluded. The full texts of 23 papers were analyzed, of which 11 with no keyword relevance and one full text of the article not available in English were excluded. The process yielded 15 articles meeting the inclusion criteria (Figure 1).

### 3.1. Studies Characteristics

Eligible articles (*n* = 15) were published between 1997 and 2021. Most of them were descriptive and cross-sectional studies [7,9,11,12,13,14,15,17,18,20]; some were both descriptive and correlational studies [5,8,10,16,20]. Thirteen out of fifteen publications focused only on nurses (number of surveyed nurses ranged from 95 to 500) [5,6,7,8,9,10,12,13,14,15,16,17,18,19]. The remaining two focused on both nurses and midwives, *n* = 133 [11] and social workers, *n* = 793 [20].

For some nurses (*n* = 80 to 285) who participated in the study, public hospital was the main and only place of employment [5,7,8,9,10,11,12,17]. There were also nurses who were employed in public hospitals (*n* = 132 to 483) and private hospitals (*n* = 39 to 352) [13,14,15,16,19].

In most publications, there was no information on specific departments at which the nurses worked, *n* = 171 to 500 [8,9,10,13,14,15,16,18,19,20]. Other studies revealed in which departments the nurses were employed, namely, at an internal medicine ward, *n* = 79 to 108 [7,12,17], surgical ward, *n* = 59 to 83 [8,12], intensive care unit, *n* = 54 to 142 [5,7,12], emergency department, *n* = 54 [7], and obstetric and pediatric wards, *n* = 80 [11].

The research instruments used in the publications to assess the hospital ethical climate were the Ethical Climate Questionnaire (ECQ) compiled by Victor and Cullen (1998) [7,9,11,14,15,16,17,18,20] and the Hospital Ethical Climate Survey (HECS) created by Olson (1995) [5,8,10,12,13,19].

Job satisfaction was assessed using the following instruments: the Minnesota Job Satisfaction Questionnaire (MSQ) (Weiss, 1967) [8,9,10,12]; the Index of Job Satisfaction (JS) (Curry, 1986) [14]; the Job Satisfaction Index (Brayfield and Rothe, 1951) [7]; the Manual for the Managerial Job Satisfaction Questionnaire (Cellucci and DeVries, 1978) [15,16,17,20]; and the Physician Job Satisfaction Scale (Williams, 1999) [20].

Detailed characteristics of the study are presented in Table 1.

### 3.2. The Influence of Ethical Climate on Perceived Job Satisfaction among Nurses Working in Health Care Institutions

#### 3.2.1. The Level of Ethical Climate and Job Satisfaction of Nurses in Health Care Units

In the study by Ulrich [20], nurses and social workers employed in hospital rated the ethical climate in the work environment as bordering or neutral but unsatisfactory. Furthermore, more than half of those surveyed indicated that there were some ethical difficulties they were helpless to deal with. Moreover, many of those surveyed indicated feelings of tension, fatigue, and depression. Lack of organizational support on ethical issues may result in nursing staff leaving the workforce. Nursing staff and social workers with higher education manifested more disappointment with their job duties. Health care professionals having a certain amount of knowledge, qualifications, and skills were able to perceive, discuss, and communicate ethical issues in their work environment. However, a difficult access or complete lack of access to resources and unsupportive ethical climate generated feelings of resentment, dissatisfaction, and moral distress manifested by, among other things, decisions made by others, feelings of guilt, or disregard for them [20]. Additionally, for Tehran nurses working in a teaching hospital [8], ethical climate was at an average level, as was job satisfaction. However, it was noted that nurses’ teamwork triggered more professional satisfaction than individual work, which could indicate the synergistic effect when working in a group. Nurses with professional degrees and those who recently started their work in hospital rated the level of ethical climate as “rather positive”. This is associated with, among other things, having higher education, skills and competencies, job satisfaction, and lower quit rates of nursing staff [13].

Factors determining the perceptions of ethical leadership among nursing staff included gender, acquired education, qualifications and skills, place of work (hospitals, outpatients along with private ones), length of service, and number of patients cared for [12]. In addition, sociodemographic variables such as ethnicity, age, the type of department where the nurses work, and their salary affected job satisfaction of the nursing staff [9].

#### 3.2.2. Types of Ethical Climate and Their Relationship to Nursing Staff Job Satisfaction

Taking into account five types of ethical climate [1], a statistically significant relationship was found between four types of ethical climate (caring, independence, rules, and professionalism) and job satisfaction. In contrast, no relationship was found between the instrumental type of ethical climate and job satisfaction [7]. There are contradictory results regarding the impact of ethical climate of rules on job satisfaction. In the study by Dinc [18], job satisfaction was significantly affected by two types of ethical climate, caring (positive) and rules (negative). On the other hand, Abadiga [9] showed that there was a positive correlation between the rules and law climate and job satisfaction which translated into the elimination of ambiguity when dealing with ethical issues and an increase in job satisfaction among nursing staff. Additionally, Karaca [11] found that respondents who were satisfied with their jobs scored statistically higher on ethical climate dimensions such as caring, independence, rules, and law. No statistically significant relationship was observed between nurses’ perception of ethical climate and their intention to leave their jobs.

In the results obtained by Goldman [17], the level of education and seniority of nurses significantly affected the perception of the ethical climate in the work environment. The greatest discrepancies were noted in the perception of the caring and independent climate which reflected negatively on perceptions of job satisfaction. Referring to the caring ethical climate, almost all the participants in the study stated that this climate was not fully practiced so the nursing staff wished to emphasize its presence more. In addition, nurses with many years of professional practice were confirmed to show more interest in instrumental and independent climate, in contrast to those with shorter seniority.

However, Tsai [16] noted that the instrumental ethical climate had a significantly negative impact on not only nurses’ overall job satisfaction but also organizational affiliation.

#### 3.2.3. Level of Ethical Climate and Job Change or Resignation among Nurses

Strong relationships are identified between perceived ethical climate and self-assessment of one’s competence and willingness to change jobs, and job satisfaction in the dimension of quality of care among nurses. The nursing staff perceiving a significantly positive ethical climate of the organization rated their own competence level as high, showed job satisfaction, and did not wish to change their place of employment [13]. Low salary in conjunction with ethical climate correlated positively with the decision to leave the profession, while a satisfactory level of organizational climate influenced staff promotions for their performance [14].

Rivaz [28] perceived that despite the friendly ethical climate, average values were recorded for the frequency rate as well as the intensity of job burnout. Moreover, the high rate of nurse turnover and poor workplace atmosphere adversely affected the quality as well as efficiency of the care services provided. Consequently, problems arose in “nurturing” an appropriate ethical climate that was essential for success [14].

#### 3.2.4. The Role of the Leader and Manager in Building and Developing an Ethical Climate

Ethical climate is not a permanent feature of a given health care unit. Its level may be in a state of flux due to, for example, changing managers who introduce new ways of managing their subordinates. In practice, nurse leaders have the responsibility of ensuring the conditions for fulfilling the organization’s mission and reconciling this with the work environment on a daily basis [17]. According to a study conducted by Jang and Özden [10,12], both the ethical climate and ethical leader had a significant impact on nurses’ job satisfaction. People-centered leadership led to higher job satisfaction because the nurses felt that their supervisors cared about them, respected them, and supported them. Nursing leaders contributed to the development of an ethical climate in the unit by putting ethical values first in their relationships with patients and staff members and followed ethical principles in the provision of care. An ethical climate promoted the alignment, support, and internalization of professional values [14]. The findings of Huang [15] showed that hospital managers could enhance elements of the professional environment such as conditions conducive to patient care, a climate based on rules and laws, normative commitment, and emotional commitment, ultimately leading to feelings of higher job satisfaction. However, this is not always the case and a lack of support from managers in the decision-making process, a lack of willingness to listen to the nurses’ representatives, and a lack of trust and respect are reported [13].

#### 3.2.5. Strategies Geared toward the Improvement of Nurses’ Ethical Climate and Job Satisfaction

In order to improve the ethical climate in health care units, there are different actions proposed, e.g., the creation of reward/motivation systems to retain nurses in the workplace [14], organization of trainings, and promotion of the idea of ethical climate among nursing staff through participation in workshops, seminars, or periodic counseling to develop leadership competencies among nurses [29].

Additionally, Aloustani [29] suggested that ethical leadership and ethical dilemma management should be included in the curriculum of future nursing students. Goldman [17] emphasized the role of the chief nurse in shaping cohesiveness strategies based on promoting an ethical workplace climate that would encourage teamwork with consideration of their most important needs. According to Jang [10] an essential determinant of nurses’ job satisfaction was ethical leaders who care, respect, and support them; therefore, health care units should consider moral competence when recruiting employees.

## 4. Discussion

Ethical climate is a fundamental element of organizational functioning that directly influences both the actions and behaviors of individuals and the community as a whole. It serves as a tool for regulating the internal nursing team, reflecting their attitudes, emotions, expectations and ways of behaving [30].

The aim of this study was to map and summarize the available research findings on ethical climate and its relationship with job satisfaction among hospital-based nursing staff. Based on the analysis of the studies conducted, a positive correlation was found between ethical climate and job satisfaction in the nursing profession.

The review of available literature shows a growing trend of interest in the issue of ethical climate of an institution such as a hospital (7 articles out of 15 were published in the last 5 years, 2016–2021) among researchers and its correlation with job satisfaction of nurses. This demonstrated the relevance and necessity of further research deepening this issue, dissemination of evidence-based research findings to both medical staff and hospital staff in leadership positions updating their existing knowledge and encouraging continuous development of ethical issues in nursing.

According to the results, the more positive the ethical climate was declared by nurses working in hospital, the higher their job satisfaction level [16]. A statistically significant relationship was also observed between the types of ethical climate (professionalism, independence, rules and laws, and caring) and job satisfaction. However, results of the studies are contradictory, in some studies the correlation was positive in some, negative.

The findings of Özden [12] showed that higher ethical climate and ethical leadership scores were recorded among nurses who were professionally fulfilled. Analogous results were obtained by Hart [31] who signaled the occurrence of a strong correlation between ethical climate and willingness to work in another health care unit. Nursing staff’s attainment of high values regarding ethical leadership and ethical climate in their work environment significantly contributed to their perception of job satisfaction.

In case of the instrumental climate, it was the least preferred by study participants and had a negative impact on job satisfaction [19]. A low level of ethical climate in hospital was pointed out to impinge on nurses’ stress burden, willingness to quit and lack of job satisfaction which in turn, resulted in the delivery of lower quality patient care [28]. Rivaz [28], Bohrani [7], Abou [14], and Numminen [13] suggested that managers of health care units should create plans to improve the ethical climate, analyze the factors that increase professional burnout, and implement preventive strategies.

Among the strategies that would contribute to an increased ethical climate, many of the authors of the reviewed publications focus on the introduction of ethics education in the workplace. On the other hand, with regard to job satisfaction, the authors propose motivational systems based on financial rewards (e.g., salary supplements).

Understanding the phenomenon of the ethical climate and skilled management of this climate in the health care unit could allow us to counteract negative phenomena quite often identified in the nurses’ workplace, i.e., decline or lack of job satisfaction, lower quality of care, occupational burnout, and finally resignation from work.

Given the results of the scoping review and the evident relationship between the ethical climate of the hospital and the job satisfaction of nurses, it is also worth to consider the introduction of quality certification of the ethical climate by external institutions, not directly related to the health care organization that could objectively assess the actual state of the ethical environment of the hospital or grant the hospital accreditation confirming that the organizations operate in accordance with the best practices in this field. This would encourage both patients and medical staff to trust the healthcare facility, and it could also influence the choice of a job by nursing graduates. It is also worth investing in ethics training of nursing managers and leaders, due to their role in creating the hospital’s ethical climate.

### Limitation of the Study

The study presented is subject to several limitations. First of all, resources from three databases were used considering only research papers written in English, and only primary sources were included into analysis. This may project a bias in the selection of the research material. Additionally, as for the scoping review [27], we did not appraise the quality of evidence included in analysis in any formal sense.

## 5. Conclusions

The scoping review of the primary sources showed that the interest of scientists in the study of the ethical climate of the hospital and its relation to job satisfaction among nurses is high. However, most of the publications come from outside Europe.

Despite the clear relationship between the ethical climate of a healthcare facility and the job satisfaction of nurses, the proposed strategies to improve the ethical climate are limited in scope. This implies new lines of research in this topic. Other areas of inquiry that result from the conducted review include the analysis of the relationship between the ethical climate of the hospital and the phenomenon of missed nursing care referred to the job satisfaction of nurses. Additionally, it would be worth getting to know the perspective of the nursing managers themselves on the issue of the ethical climate of a healthcare facility and its relation to the job satisfaction of nurses, as well as the perspective of other members of the therapeutic team.

## Figures and Tables

**Table 1:** Main characteristics of studies included.

Author(s) /Year/ Country	Method and Research Design	Sample	Research Instruments	Relevant Main Findings
Joseph et al., 1997 (USA)	Descriptive, cross-sectional study	Nurses (*n* = 114).	(1) Ethical Climate Questionnaire; (2) Manual for the Managerial Job Satisfaction Questionnaire	Among the different dimensions of job satisfaction, the respondents were most satisfied with their job (mean = 3.18). Professional, instrumental, and independent climate did not affect job satisfaction. Caring climate significantly affected satisfaction with overall job satisfaction.
Ulrich et al., 2007 (USA)	Descriptive, cross-sectional, correlational study	Nurses and social workers (*n* = 1215).	(1) Physician Job Satisfaction Scale; (2) Ethic Stress Questionnaire; (3) Hospital Ethical Climate Scale	Ethical climate in nurses work environment was higher than neutral mean score of 97.3 (median 98; range 35–130; SD = 14.4). Perceived ethical climate and adequacy of institutional ethical sources, years in current position, impact of feeling respected as a team member and identity with the institution’s mission negatively correlate with intention to leave and positively with job satisfaction. Ethical climate partially mediates the relationship between ethical stress and job satisfaction, while job satisfaction completely mediates the relationship between ethical stress and intention to leave.
Tsai et al., 2008 (Taiwan)	Descriptive, cross-sectional, correlational study	Nurses (*n* = 352)	(1) Ethical Climate Questionnaire; (2) Managerial Job Satisfaction Questionnaire	Caring climate has a significant and positive relationship with nurses’ satisfaction with salary, the job itself, and overall job satisfaction. Independent climate significantly and positively affects nurses’ satisfaction with supervisors and overall job satisfaction but has no effect on any component of organizational commitment (OC). The law-and-rules climate does not significantly and positively influence any of the aspects of job satisfaction. Principle climate significantly and positively determined satisfaction with supervisors, co-workers, salary and overall job satisfaction. Instrumental climate negatively and significantly affects overall job satisfaction, promotion satisfaction, and overall OC.
Goldman et al., 2010 (Israel)	Descriptive, cross-sectional study	Nurses (*n* = 95)	(1) Ethical Climate Questionnaire; (2) Managerial Job Satisfaction Questionnaire	The discrepancy in perceptions of caring and independent climates indicate a decrease in nurses’ job satisfaction, while perceptions of an actual climate of caring and service positively affect all aspects of job satisfaction. It is recommended that training programs should be constructed to emphasize the ethics of nursing practice and to assist nurses in developing an ethical framework and guiding nursing staff in dealing with ethical dilemmas.
Huang et al., 2012 (Taiwan)	Descriptive, cross-sectional study	Nurses (*n* = 352)	(1) Ethical Climate Questionnaire; (2) Managerial Job Satisfaction Questionnaire; (3) Organizational Commitment Questionnaire; (4) Organizational Citizenship Behavior Questionnaire	Findings indicate that hospital managers can enhance organizational climate elements such as an atmosphere conducive to care; a climate based on laws, codes and rules; satisfaction with co-workers; affective commitment; and normative commitment, which increase organizational citizenship behaviors while preventing instrumental and fixed commitment climates that decrease them.
Borhani et al., 2012 (Iran)	Descriptive, cross-sectional study	Nurses (*n* = 275)	(1) Ethical Climate Questionnaire; (2) Job Satisfaction Index	The results showed a positive correlation between ethical climate such as professionalism, rules, caring, independence and job satisfaction, while no correlation was found between instrumental climate and job satisfaction. Hospital executives can help improve nurses job satisfaction through ethics training programs to create a collaborative system and culture that enhances team spirit among staff.
Joolaee et al., 2013 (Iran)	Descriptive, cross-sectional, correlational study	Nurses (*n* = 210)	(1) Hospital Ethical Climate Survey; (2) Minnesota Job Satisfaction Questionnaire	The mean and standard deviation of the results of the Olson climate questionnaire was 3.36 ± 0.69, and the mean and standard deviation of job satisfaction among the nurses was 3.17 ± 0.63. A significant positive relationship was found between the ethical climate in hospital and the level of job satisfaction among the nurses, (r = 0.39, *p* ≤ 0.001). The type of assigned tasks, income level and work shift were significantly related to job satisfaction among the demographic variables. The study showed that the more favorable the ethical climate, the higher the level of job satisfaction reported by the nurses.
Numminen et al., 2015 (Finland)	Descriptive, cross-sectional, correlational study	Nurses (*n* = 318)	(1) Hospital Ethical Climate Survey; (2) Nurse Competence Scale	Strong associations were found between perceived ethical climate and self-assessment of competence, intention to change jobs, and job satisfaction in the context of quality of care. There is also a need to know the views of newly graduated nurses on the factors that act as enhancers or hindrances to the development of a positive ethical climate. Interventions, continuing education courses and discussions to promote a positive ethical climate should be developed for managers, nurses, etc.
Abou et al., 2017 (Egypt)	Descriptive, cross-sectional study	Nurses (*n* = 500)	(1) Ethical Climate Questionnaire; (2) Survey of Perceived Organizational Support; (3) Organizational Commitment Questionnaire; (4) Index of Job Satisfaction; (5) Turnover Intention scale	Findings revealed positive significant correlations between nurses’ perceptions of overall ethical work climate and each of the perceived factors: organizational support, commitment, and job satisfaction. In contrast, negative significant correlations were found between intention to change jobs and each of these variables.
Dinc et al., 2017 (Bosnia and Herzegovina)	Descriptive, cross-sectional study	Nurses (*n* = 171)	(1) Ethical Climate Questionnaire; (2) Organizational Commitment Questionnaire; (3) Job Satisfaction Index	Path analysis revealed that ethical climate elements such as rules and caring significantly and positively influenced overall job satisfaction. In contrast, in the second analysis overall job satisfaction and the rules component of ethical climate were found to significantly affect normative commitment. In contrast, caring and overall job satisfaction significantly affected affective commitment.
Karaca et al., 2018 (Turkey)	Descriptive, cross-sectional study	Nurses (*n* = 80), midwives (*n* = 133)	Ethical Climate Questionnaire	A significant difference was found between job satisfaction of nurses and midwives and perception of ethical climate. A significant difference was found between nurses and midwives job satisfaction and perceptions of ethical climate, with the strongest relationship shown in the caring dimension.
Asgari et al., 2019 (Iran)	Descriptive-correlation study	Critical care nurses (*n* = 142)	(1) Moral Distress Scale–Revised; (2) Hospital Ethical Climate Survey; (3) Job Satisfaction Index	The mean scores of the critical care nurses for moral distress, ethical climate and job satisfaction were 87.02 ± 44.56, 3.51 ± 0.53, and 62.64 ± 9.39, respectively. No significant relationships were observed between moral distress and job satisfaction, the relationship between ethical climate and job satisfaction was statistically significant (*p* < 0.05).
Özden et al., 2019 (Turkey)	Descriptive, cross-sectional study	Nurses (*n* = 285)	(1) Hospital Ethical Climate Survey; (2) Ethical Leadership Scale, (3) Minnesota Job Satisfaction Questionnaire	The levels of ethical leadership (mean score 59.05 ± 14.78), ethical climate (mean score 92.62 ± 17), and job satisfaction (mean scores 62.15 ± 13.46) of the nurses were moderate, and there was a positive relationship between them. Nurses’ perceptions of ethical leadership are influenced by their educational status, workplace, and seniority. The correlation between the nurses’ mean scores for ethical leadership and ethical climate was moderately positive and statistically significant (r = +0.625, *p* = 0.000). The correlation was weak but statistically significant between ethical leadership and job satisfaction mean scores (r = +0.461, *p* = 0.000). This study showed a moderate, positive and statistically significant correlation between ethical climate and job satisfaction mean scores (r = +0.603, *p* = 0.000) among nurses.
Jang et al., 2019 (South Korea)	Descriptive, correlational study with a convenience sample	Nurses (*n* = 263).	(1) Hospital Ethical Climate Survey; (3) Ethical Leadership Scale; (4) Minnesota Job Satisfaction Questionnaire; (5) Ethical Leadership at Work Questionnaire	Job satisfaction was positively correlated with ethical climate and ethical leadership. Ethical climate in relation to hospitals and people-oriented leadership were influential factors in the level of job satisfaction among nurses.
Abadiga et al., 2019 (Ethiopia)	Institutional based cross-sectional study	Nurses (*n* = 266).	(1) Hospital Ethical Climate Survey; (2) Minnesota Job Satisfaction Questionnaire	The study showed moderate level of hospital ethical climate (mean 53.4) and moderate level of job satisfaction (mean 51.3%) among nurses. Law and code climate significantly influenced job satisfaction (β = 1.53, *p* = 0.000). Care climate also had a significant influence on nurses’ job satisfaction (β = 0.99, *p* = 0.000). Independence climate had a significant influence on job satisfaction (β = 0.62, *p* = 0.041). Control climate and instrumental climate had no significant influence on job satisfaction (β = 0.380, *p* = 0.409 and β = −0.208, *p* = 0.290, respectively). Poor ethical climate in hospital worsened the level of nurses’ job satisfaction.

## Data Availability

Not applicable.
